# Use of Phosphorus-32 Microparticles for Downstaging Locally Advanced Pancreatic Cancer to Resection: A Case Report

**DOI:** 10.7759/cureus.108579

**Published:** 2026-05-10

**Authors:** Fernando Pereira, Ignacio Juez, Virginia Peiro, Antonio Guardiola, Covadonga del Riego, Pablo Haro

**Affiliations:** 1 Department of Surgery, Hospital Universitario de Fuenlabrada, Madrid, ESP; 2 Department of Medical Oncology, Hospital Universitario de Fuenlabrada, Madrid, ESP; 3 Department of Nuclear Medicine, Hospital Universitario de Fuenlabrada, Madrid, ESP; 4 Department of Gastroenterology and Gastrointestinal Endoscopy, Hospital Universitario de Fuenlabrada, Madrid, ESP; 5 Department of Radiology, Hospital Universitario de Fuenlabrada, Madrid, ESP

**Keywords:** 32p microparticles, brachytherapy, case report, locally advanced, pancreatic cancer

## Abstract

Unresectable locally advanced pancreatic cancer (LAPC) is managed with first-line chemotherapy or induction chemotherapy with consolidative chemoradiotherapy. Phosphorus-32 (^32^P) microparticles serve as a brachytherapy device, approved for use in LAPC, that has the advantage of delivering radiation directly to the tumour while reducing the risk of damage to nearby tissue or organs. The case of a 72-year-old woman with an unresectable pancreatic adenocarcinoma in the head of the pancreas with long-term survival following downstaging to resection with ^32^P microparticles is presented here. The patient received first-line gemcitabine+nab-paclitaxel chemotherapy, with a partial response occurring during the first three cycles of chemotherapy; however, the tumour remained unresectable and subsequently increased in size after a justified delay in chemotherapy. ^32^P microparticle implantation was conducted as planned at the end of cycle 4, and no device- or procedure-related adverse events were observed. Gemcitabine+nab-paclitaxel chemotherapy was continued, and computed tomography (CT) imaging during cycle 6 (6.8 months after diagnosis) revealed stabilisation of tumour size and a slight decrease in the density of the pancreatic mass. The surgical team determined that surgery could be feasible at this stage. Surgical resection by an open Whipple procedure was successfully completed 7.9 months after diagnosis. As of January 2026, the patient remains alive without evidence of disease 50 months after diagnosis and 42 months post-resection. This case study shows that the addition of ^32^P microparticles to first-line gemcitabine+nab-paclitaxel chemotherapy, in a patient with unresectable LAPC, is associated with not only disease stabilisation but also conversion to surgical resection, with no associated radiation-related adverse events.

## Introduction

Unresectable locally advanced pancreatic cancer (LAPC) accounts for approximately 30% of pancreatic cancer presentations [1] and has a poor prognosis with a median survival of around 13.3 months [2]. Approximately 13% of patients with unresectable LAPC are downstaged to resection following systemic chemotherapy, and these patients experience significantly longer survival compared to patients who do not convert to resectability [3].

Guidelines recommend first-line chemotherapy or induction chemotherapy with consolidative chemoradiotherapy; preferred first-line chemotherapy regimens are gemcitabine+nab-paclitaxel or FOLFIRINOX (leucovorin calcium (folinic acid)), fluorouracil, irinotecan hydrochloride, and oxaliplatin) [4]. Combining systemic chemotherapy with radiotherapy can improve local disease progression, pain control, performance status, and quality of life, and in a neoadjuvant setting, it may also increase conversion to resectability [5]. Brachytherapy, a form of radiotherapy, involves implanting radioactive material inside or in close proximity to a tumour to target cancer cells directly. Due to the location of the pancreas, there is a risk of radiation damage to nearby radio-sensitive tissues and organs with external radiotherapy; therefore, a more locally directed brachytherapy using endoscopic ultrasound (EUS)-guided fine-needle injection [6] can deliver radiation directly to the tumour and reduce the risk of collateral damage in unresectable LAPC [7,8]. Phosphorus-32 (^32^P) microparticles (OncoSil™, OncoSil Medical, Sydney, Australia) serve as a brachytherapy device, approved for use in unresectable LAPC, that implants the required activity of beta radiation-emitting ^32^P microparticles into pancreatic tumours via EUS guidance to deliver an absorbed dose of 100 Gy to the tumour. The PanCO study suggested that EUS-guided ^32^P microparticle implantation has an acceptable safety profile and that there are clinically relevant benefits of combining ^32^P microparticles with standard-of-care systemic chemotherapy for patients with unresectable LAPC [9].

Here, we present the case report of a female patient diagnosed with LAPC, treated with first-line chemotherapy, who had eventual downstaging to resection following the addition of ^32^P microparticles.

## Case presentation

**Figure 1:**
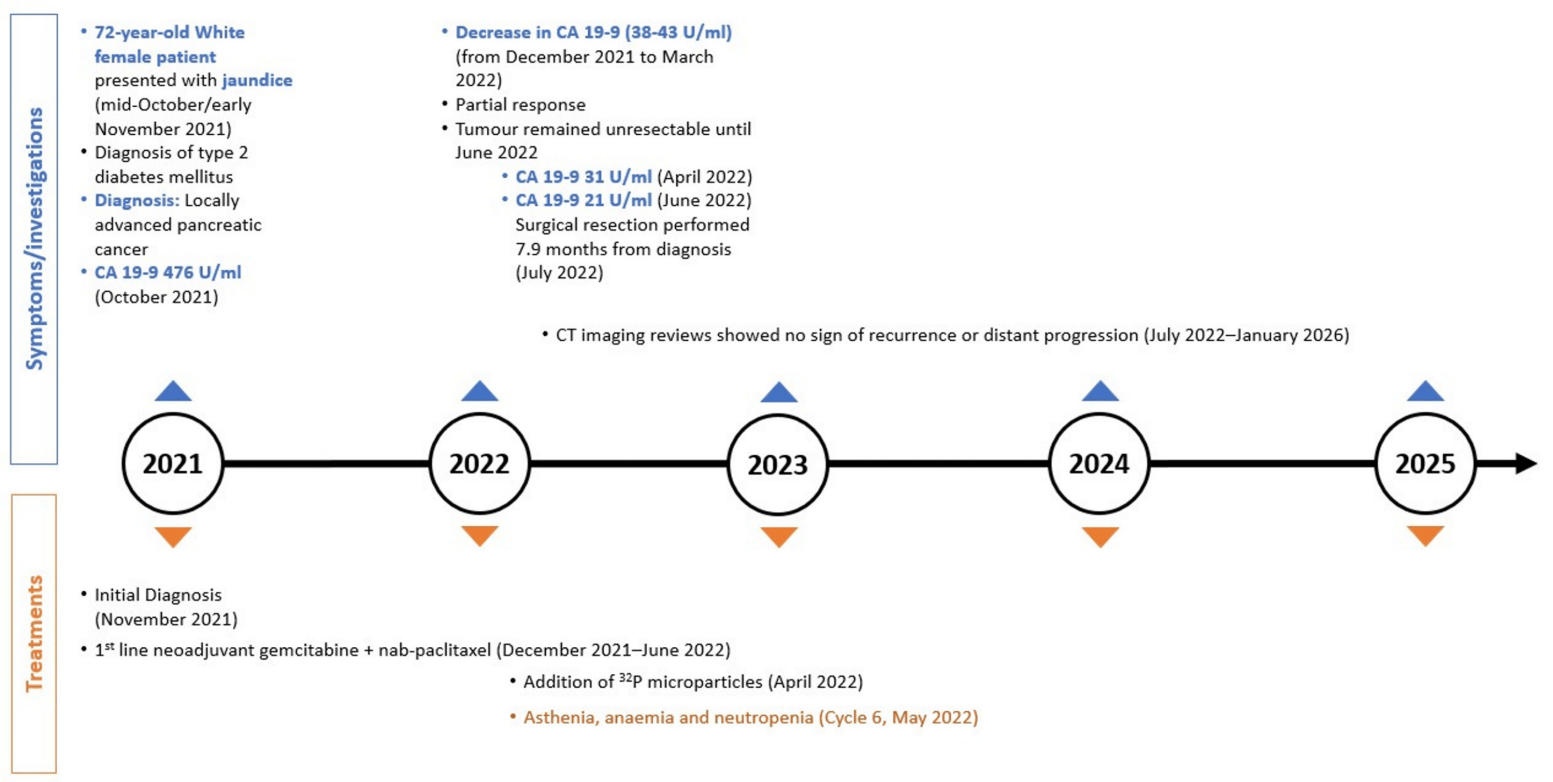
Timeline of treatments and investigations Upper limit of normal for CA 19-9: 37 U/ml CA 19-9: carbohydrate antigen 19-9; CT: computed tomography

An overview of the case is provided in Figure 1. A 72-year-old White woman with a 10-year history of controlled paranoid schizophrenia presented to her general practitioner with jaundice in October 2021. She was referred to the hospital for examination and diagnosis. The patient was found to have elevated blood glucose (313 mg/dl), bilirubin (10.85 mg/dl), and carbohydrate antigen (CA) 19-9 (476 U/ml; upper limit of normal (ULN) 37 U/ml) levels. Elevated glucose levels indicated type 2 diabetes mellitus.

**Figure 2:**
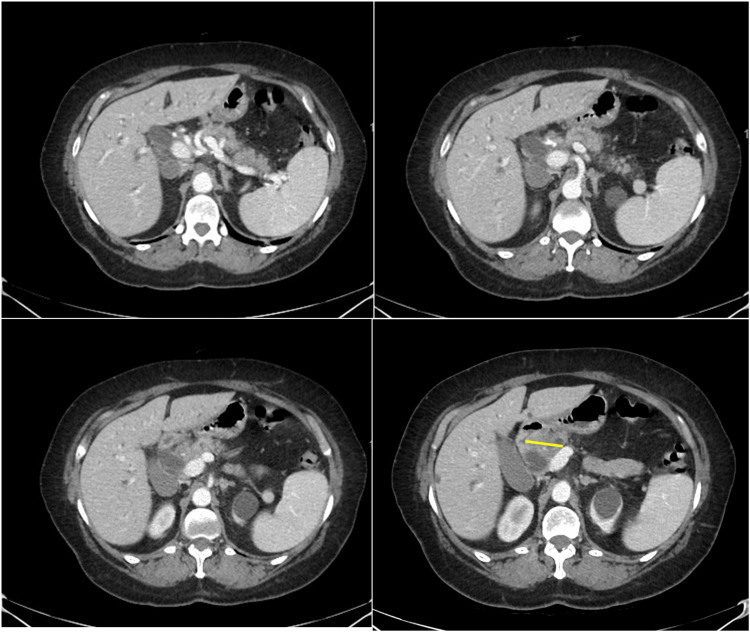
CT imaging of the pancreatic tumour at diagnosis. The yellow bar indicates the longest tumour diameter CT: computed tomography

Computed tomography (CT) imaging on 16th November 2021 suggested pancreatic adenocarcinoma (PDAC) in the head of the pancreas. The CT imaging showed a very poorly defined lesion, with longest diameters of 29×15 mm that projected towards the hepatic hilum with a soft tissue cuff that completely surrounded the hepatic artery (HA) for a length of 20 mm, including its bifurcation into right and left branches (Figure 2). Medially, the mass contacted the most cranial portion of the superior mesenteric vein (SMV) and the origin of the portal vein (PV), without signs of infiltration. The tumour was determined to be unresectable (according to the National Comprehensive Cancer Network (NCCN) guidelines, Version 1.2022 [10]), LAPC T4N0M0 stage III (American Joint Committee on Cancer (AJCC), 8th edition). No genetic testing was performed as per hospital protocol, as this was the first case of PDAC in the family. There were no other challenges affecting either access to testing or any financial or cultural issues with this patient.

Endoscopic retrograde cholangiopancreatography (ERCP) was performed on 17th November 2021 to address the patient's rising bilirubin level (14 mg/dl). Stenosis of the distal common bile duct was observed at this time, caused by the underlying neoplasm, and a 60×10 mm uncoated metallic stent was inserted during the ERCP procedure with no complications. Positron emission tomography (PET)-CT imaging on 22nd November 2021 demonstrated minimal fluorodeoxyglucose (FDG) uptake and metabolism by the tumour, without locoregional or distant dissemination. Biopsy by endoscopic ultrasound-guided fine needle aspiration (EUS-FNA) on 30th November 2021 confirmed PDAC. An Eastern Cooperative Oncology Group performance status (ECOG PS) of 1 was noted for the patient on 9th December 2021.

Due to the patient's general health status and presence of comorbidities, she was considered unlikely to be able to tolerate FOLFIRINOX; therefore, standard-dose gemcitabine+nab-paclitaxel chemotherapy (total administered doses per 28-day cycle: 5016 mg and 495 mg, respectively) was commenced at 0.9 months from diagnosis (13th December 2021). CA 19-9 levels showed a rapid decrease after biliary drainage and the first cycles of chemotherapy, with levels (38-43 U/ml) just above the ULN (37 U/ml) from cycle 1 to cycle 3 (measures were taken on the first day of each cycle and as needed). There were no adverse events related to chemotherapy in the first three cycles, and no dose changes were required.

**Figure 3:**
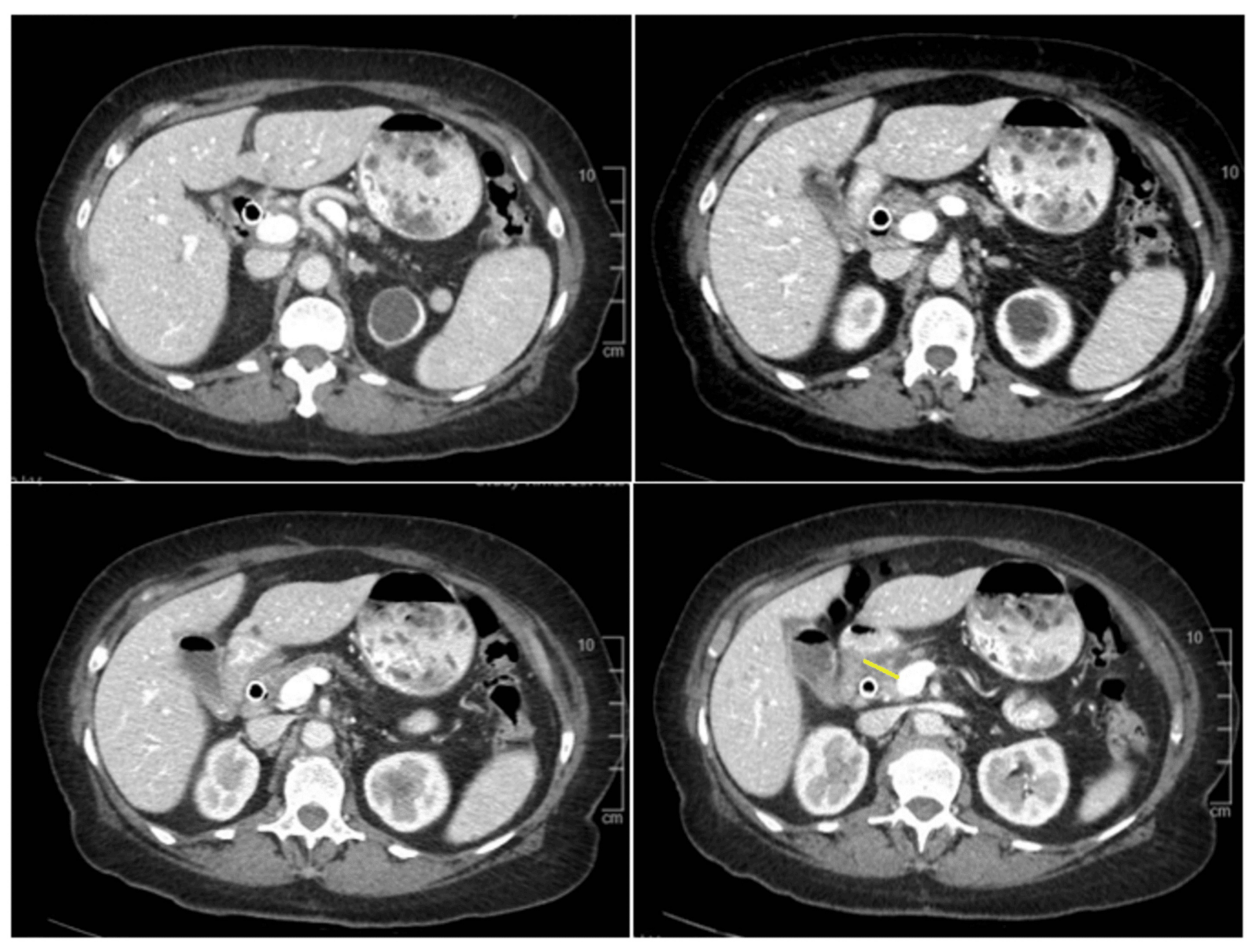
CT imaging of the pancreatic tumour at 3.8 months from diagnosis. The yellow bar indicates the longest tumour diameter CT: computed tomography

CT imaging on 10th March 2022, which was 3.8 months after diagnosis and after three cycles of gemcitabine+nab-paclitaxel chemotherapy, showed a partial response (PR) by RECIST Version 1.1, with a 30% reduction in the longest tumour diameter from the first CT scan (Figure 3). Resectability was re-assessed, and the tumour remained unresectable, with encasement of the common and proper HA (CHA; PHA) to its distal branches still observed and contact with the PV without fatty plane separation. In addition, there was concern regarding possible liver metastases in the CT scan, and chemotherapy was temporarily delayed to enable magnetic resonance imaging (MRI) (at the end of March 2022), which ruled out the existence of hepatic metastases. Standard-dose gemcitabine+nab-paclitaxel chemotherapy was resumed from cycle 4 (initiated 28th March 2022), following an approximately three-week delay.

Given the risk of radiation damage to nearby tissues and organs and the continued association of the tumour with the CHA and PHA, it was determined that it would be beneficial to use brachytherapy to deliver radiation directly to the tumour. As such, the addition of ^32^P microparticles was planned for the end of cycle 4, with the intent of downstaging the tumour to enable surgical resection. Additional unscheduled CT imaging was performed on 21st April 2022 (5.2 months from diagnosis) to measure the tumour volume for the calculation of the ^32^P microparticle activity and volume to be implanted. This revealed a tumour volume of 9.9 ml. CA 19-9 (31 U/ml; ULN 37 U/ml) level remained within the normal range. Vascular involvement remained as previously described. ^32^P microparticle implantation (26th April 2022, 5.3 months from diagnosis of the primary tumour) was performed under EUS guidance via the gastric wall. Two deposits were implanted in the tumour close to the involved vessels (first 25% activity in distal and then 75% proximal after retracting; 5.2 MBq ^32^P microparticle was implanted). Targeting of the implantation was planned and directed by the nuclear medicine physician, in contact with the endoscopist. No difficulties were experienced during ^32^P microparticle implantation, the localisation of which was confirmed four hours post-implantation by single-photon emission computed tomography-CT (SPECT-CT). No adverse events were experienced during the implantation procedure or prior to discharge, and no adverse events were subsequently associated with ^32^P microparticles or the implantation procedure, suggesting that the treatment was well tolerated.

**Figure 4:**
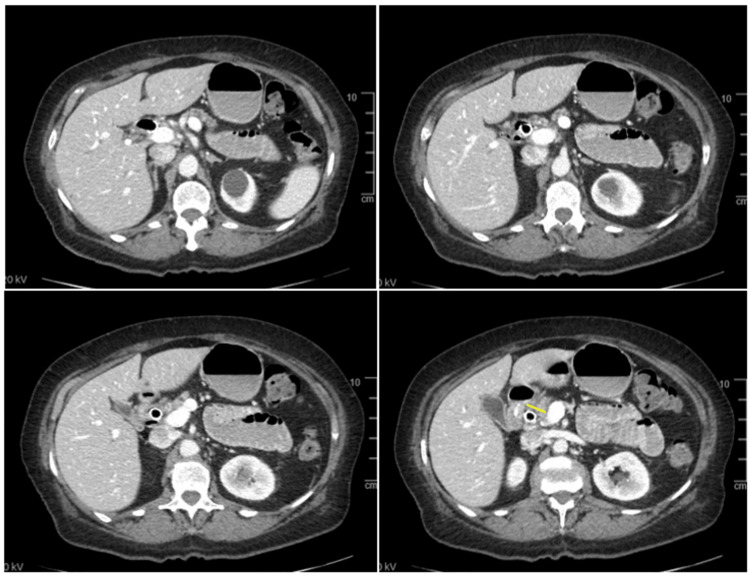
CT imaging of the pancreatic tumour at 6.8 months from diagnosis. The yellow bar indicates the longest tumour diameter CT: computed tomography

Gemcitabine+nab-paclitaxel chemotherapy was continued with cycle 5 (initiated 4th May 2022), with no treatment delays as the implantation procedure was conducted in the resting week of the previous chemotherapy cycle. However, the dose intensity was reduced in cycle 6 (total administered doses per 28-day cycle: 3344 mg and 230 mg, respectively; cycle started on 31st May 2022) due to grade 2 asthenia, anaemia (despite iron supplementation), and neutropenia. CT imaging on 9th June 2022 (6.8 months from diagnosis) during chemotherapy cycle 6 revealed stabilisation of tumour size with a longest diameter of 5.2 cm (Figure 4). However, compared to the CT imaging 3.8 months after diagnosis, there was a slight decrease in the density of the pancreatic mass as well as a significant reduction in tumour volume to 4 ml. A soft tissue component persisted around the HA bifurcation, with similar involvement of the SMV/PV. No distant disease was observed, and CA 19-9 levels were within the normal range at 21 U/ml (ULN 37 U/ml). PET-CT imaging was not repeated due to the minimal FDG uptake at baseline. The surgical team determined that surgery could be feasible at this stage, with the possibility of having to resect and reconstruct the HA, with moderate surgical risk due to an acceptable baseline health status in a fragile patient who could walk accompanied for one hour a day and climb three flights of stairs before needing to rest. Surgical resection was performed on 14th July 2022 (7.9 months from diagnosis) by an open Whipple procedure (pancreaticoduodenectomy) with pancreaticogastrostomy. The surgical team noted a difficult separation of the HA from the tumour (due to the fibrotic tissue surrounding it) and SMV/PV, needing SMV/PV/splenic vein clamping and sharp dissection of the venous and arterial walls, but without the need for vascular resection. In addition to standard lymphadenectomy for a Whipple procedure in pancreatic cancer (stations 5, 6, 8a, 12b, 12c, 13a, 13b, 14a, 14b, 17a, and 17b), lymphadenectomy for stations 8p, 11p, 12a, 12p, and 14p was also included. Pathology on the resected specimen confirmed PDAC, with a residual tumour size with a longest diameter of 0.7 cm, moderate histological differentiation and pT1b N1 (one of 11 nodes was positive in station 13), negative lymphovascular infiltration (LV0), negative perineural infiltration (Pn0), and R0 margins (TNM, 8th edition) without any malignancy in the HA margins. This was determined to be a pathological partial response to treatment (grade 2; modified Ryan).

On 25th July 2022 (11 days after surgery), the patient was transferred to the intensive care unit (ICU) for close monitoring due to sepsis of abdominal origin. She had moderate disorientation, elevation of creatinine (1.5 mg/dl), procalcitonin (48 ng/ml), and lactate (5.9 mmol/l), thrombopenia (57,000/µl), purulent right drainage, *Enterobacter*-positive blood culture (three of four samples), and CT showing small non-drainable collections. The patient made a good recovery with haemodynamic support and antibiotics, being discharged from the ICU on 28th July 2022. She was potentially able to be discharged from the hospital on 2nd August 2022 (19 days after surgery), but social care attendance was unavailable; therefore, discharge finally occurred on 25th August 2022.

The patient was unable to receive adjuvant chemotherapy due to ongoing absence of family or social support. Post-surgical CT imaging (performed every five months thereafter until January 2026) showed no sign of recurrence or distant progression with tumour markers within the normal range throughout follow-up. As of January 2026, the patient remains alive without evidence of disease 50 months post-diagnosis (42 months post-resection). She takes pancreatic enzyme supplements (Kreon®) with no exocrine insufficiency symptoms and without worsening of her diabetes, which is managed using metformin and sitagliptin, and has a good performance status (ECOG PS 0).

Patient perspective

While this patient had other conditions, including paranoid schizophrenia, and had to undergo major surgery (pancreaticoduodenectomy) at an advanced age, she noted improvements to her quality of life (even though quality of life was not formally assessed). This has been maintained for over three years post-diagnosis of LAPC, particularly now that the patient remains alive without evidence of disease. The patient has no exocrine insufficiency symptoms using pancreatic supplements and has no worsening of her diabetes. She was aware of the poor prognosis of PDAC, and the side effects she experienced during her treatment did not detract from the increased hope gained by the success of her treatment.

## Discussion

In this case report, we describe a female patient diagnosed with LAPC, who was downstaged to resection following the addition of ^32^P microparticles to gemcitabine+nab-paclitaxel chemotherapy that had provided an initial response but with an increase in tumour size at 5.2 months after a justified delay in chemotherapy.

Many of the key prospective clinical trials that recruited patients with LAPC included patients with metastatic pancreatic cancer and patients with LAPC that was unresectable or borderline resectable. Hence, there are limited prospective data on the survival of patients with unresectable LAPC to compare with this current case study. In phase II studies and real-world analyses, the median overall survival (OS) following chemotherapy with gemcitabine+nab-paclitaxel has ranged from 12.5 to 18.8 months [11-13]. The case reported here, in which ^32^P microparticle implantation was combined with gemcitabine+nab-paclitaxel chemotherapy, resulted in considerably longer survival than these previous trials with gemcitabine+nab-paclitaxel alone. Indeed, while treatment with ^32^P microparticle implantation in addition to chemotherapy has not been compared directly with other standard therapies in the setting of prospective randomised controlled studies, a propensity score-weighted landmark analysis showed significantly longer survival with ^32^P microparticle implantation plus chemotherapy than with chemotherapy alone (median OS 19.7 and 12 months, respectively; p=0.002) [14]. Furthermore, combining ^32^P microparticle implantation with chemotherapy agents aims to maximise the antitumoral effects to shrink pancreatic tumours, and this may increase the rate of surgical resection and minimise the risk of local recurrence [9]. Patients undergoing surgical resection after neoadjuvant chemotherapy have significantly better survival than patients who do not undergo resection (median OS 35.3 months versus 16.3 months, respectively) [15]. In the PanCO study, 10 patients underwent surgical resection after tumour downstaging (nine received gemcitabine+nab-paclitaxel and one received FOLFIRINOX), with six remaining alive and five of those patients without disease recurrence at the end of the study, 26.4-35.3 months post-enrolment [9,16]. The long-term survivor reported in this case study remains alive without evidence of disease at 50 months post-diagnosis (42 months post-resection), comparing favourably with these previous published reports. The patient underwent a rapid drop in CA 19-9 levels after biliary drainage and the first cycles of chemotherapy, which has been proposed as an indicator of long-term survival in some studies [17]. The extended survival observed in this case is particularly noteworthy given that, prior to ^32^P microparticle implantation, factors such as an increase in tumour size and the presence of comorbidities that precluded more effective chemotherapy options, such as FOLFIRINOX, were clearly indicative of a likely poor prognostic outcome for this patient.

Encouragingly, the addition of ^32^P microparticle treatment to systemic gemcitabine+nab-paclitaxel chemotherapy was well tolerated by the patient, and side effects were low grade (asthenia, anaemia, and neutropenia in association with gemcitabine+nab-paclitaxel). No adverse events in association with ^32^P microparticles occurred.

Limitations of this report are those common to case studies, including a lack of generalisability to the wider population and that as patient treatment took place in a real-world setting, variables could not be controlled for and not all outcomes were assessed regularly.

## Conclusions

This patient, diagnosed with unresectable LAPC, has achieved an extraordinary survival of 50 months post-diagnosis, with ^32^P microparticles implanted 5.3 months after diagnosis of the primary tumour, despite having some unfavourable prognostic indicators. This case study shows that the addition of ^32^P microparticles to first-line gemcitabine+nab-paclitaxel chemotherapy, in a patient with unresectable LAPC, is associated with disease stabilisation and potentially conversion to surgical resection. This combination therapy was well tolerated in this patient, and future research is needed to determine factors that may predict long-term survival in patients with unresectable LAPC receiving ^32^P microparticles.
